# The Prevalence of Anemia and its Association with Depressive Symptoms among Older Adults in North of Iran

**Published:** 2018-12-03

**Authors:** Seyed Reza Hosseini, Ali Zabihi, Seyed Hesameddin Ebrahimi, Seyedeh Roghayeh Jafarian Amiri, Farzan Kheirkhah, Ali Bijani

**Affiliations:** ^1^ Social Determinants of Health Research Center, Health Research Institute, Babol University of Medical Sciences, Babol, Iran; ^2^ Nursing Care Research Center, Health Research Institute, Babol University of Medical Sciences, Babol, Iran; ^3^ Student Research Committee, Babol University of Medical Sciences, Babol, Iran; ^4^ Department of Nursing, Faculty of Nursing and Midwifery, Babol University of Medical Sciences, Babol, Iran; ^5^ Department of Psychiatry, Faculty of Medicine, Babol University of Medical Sciences, Babol, Iran

**Keywords:** Anemia, Depression, Older adults

## Abstract

**Background:** Anemia is the most prevalent blood disorder in older adults which can have negative effects on the quality of life and lead to the onset of depressive symptoms. We aimed to determine the prevalence of anemia accompanied by depression among older people in the city of Amirkola, north of Iran.

**Study design:** A cross-sectional study.

**Methods:** Overall, 1616 older people aged 60 and over (883 males and 733 females) were enrolled in the city of Amirkola, north of Iran since 2011. To diagnose anemia we used WHO criterion which is a hemoglobin value of less than 12 and 13 g/dl in women and men, respectively. We also used Geriatric Depression Scale (GDS) to detect the presence of depression symptoms. The data were analyzed using SPSS version18.0 and statistical tests.

**Results:** The prevalence of anemia was 19% (302 out of 1616 participants). The prevalence of anemia in women was 20.3% and in men was 17.9%. In people with and without depressive symptoms it was 23.2% and 15.8%, respectively. The mean hemoglobin level in people with and without depressive symptoms was 13.29 ±1.63 and 13.96 ±1.50, respectively (*P*<0.001). According to logistic regression model, depressive symptoms were most highly related to gender (OR=3.67; 95% CI: 2.80, 4.81) and besides that smoking, Mini-Mental Estate Examination (MMSE), diabetes and anemia (OR=1.46; 95% CI: 1.09, 1.95) were related to depression.

**Conclusion:** Significant prevalence of anemia and direct association with depressive symptoms in the elderly reflect the need for proper planning for prevention interventions, accurate and continuous screening of these diseases.

## Introduction

In most countries, especially in developing cases, the ratio of aging population is increasing rapidly ^1, 2^. Medical advances and health policies in countries like Iran, which is changing its lifestyle to the western lifestyle, lead to an increase in population of people aged 60 yr and over^3^. Regarding the international estimates, from 2040 onward, the population of older adults in Iran will grow faster than other parts of the world and even the global average^4^.

Anemia is a major health concern among older adults and usually develops due to different causes including chronic inflammatory diseases, chronic kidney disease, nutrient deficiencies, iron deficiency and unknown causes^5^. WHO defines anemia as a hemoglobin level of less than 12 g/dl in women and less than 13 g/dl in men^6^.

In older adults, anemia is caused by underlying diseases such as cancer, renal diseases, infectious diseases accompanied by heart diseases. These comorbidities have negative effects on the quality of life and lead to the onset of depressive symptoms^7^. The negative impacts of anemia are higher in older adults associated with difficulties such as decreased quality of life, functional limitations, increased risk of developing dementia, fatigue, increased risk of having a fall and increased depressive symptoms^8-11^.

Depression is a common disorder in older adults accompanied by increased risk of mortality and pathogenicity and also delays or fails the recovery from diseases^12^. The underlying mechanism of the relationship between a low hemoglobin level and depressed mood requires clarification. There have been different studies on the association between anemia and depression throughout the world^7,10^. However, the results were not constants^10,11,13^. On the other hand, no study has been made on the prevalence of anemia in the population-based survey in Iran.

Therefore, the present study was conducted with the purpose of investigating the prevalence of anemia and its association with depression in older adults in the city of Amirkola, north of Iran.

## Methods

This research was approved by the ethics committee of Babol University of Medical Sciences (MUBABOL.REC.1389.4). This cross-sectional study was a part of Amirkola Health and Ageing Project (AHAP) (registration number: 892917) conducted as a cohort study on all older adults aged 60 yr and over in the city of Amirkol in north of Iran since 2011^4^. This city has two healthcare centers which have the list of all older adults of the city. First, we contacted the participants by going to their houses or through telephone and gave them the necessary information about the project and invited them to take part in the study. Then after filling the demographic questionnaires, the participants were asked to be fast and visit the research center in the next morning for medical tests. Finally, the rest of the questionnaires were filled and complementary examinations were performed.

In order to take blood tests, we drew a sample of venous blood and performed a complete blood count using Sysmex XT-1000i made in United States. The amount of ferritin was measured using Diametra kits made in Italy and ELISA technique. A normal level was defined as a ferritin level of 20-400 ng/ml for men and 8-350 ng/ml for women. Serum iron and total iron-binding capacity (TIBC) were measured using Pars Azmoon kits made in Iran and the colorimetric method performed by Roche/Hitachi 902 made jointly by Germany and Japan. The normal amount of serum iron was considered as 35-168 µg/dl for men and 22-135 µg/dl for women and a TIBC of 230-440 µg/dl was defined as normal. Anemia was diagnosed based on WHO criterion which defines it as a hemoglobin value of less than 12 g/dl in women and less than 13 g/dl in men^6^.

The presence of depressive symptoms was investigated using Geriatric Depression Scale (GDS) which is a 15-item questionnaire and the patients can be divided into several groups based on their cumulative scores. The severity is qualified using the following cutoff: 0-4 normal, 5-8 mildly depressed, 9-11 moderately depressed and 12-15 severely depressed^15^. Elders with a score of five or more were referring to the psychiatric clinic for further investigation. GDS is a validated questionnaire in the Iranian population^16^. Cronbach’s alpha for GDS was reported 0.81 in this study.

After completion of data collection, data coding was performed using Microsoft Excel and data analysis was performed using SPSS ver. 18.0 (Chicago, IL, USA) as well as statistical tests including Chi-Square, ANOVA and logistic regression. Then, the prevalence of anemia was calculated and its association with demographic information and risk factors was investigated and a *P*-value of less than 0.05 was considered to be statistically significant.

## Results

**Figure 1:**
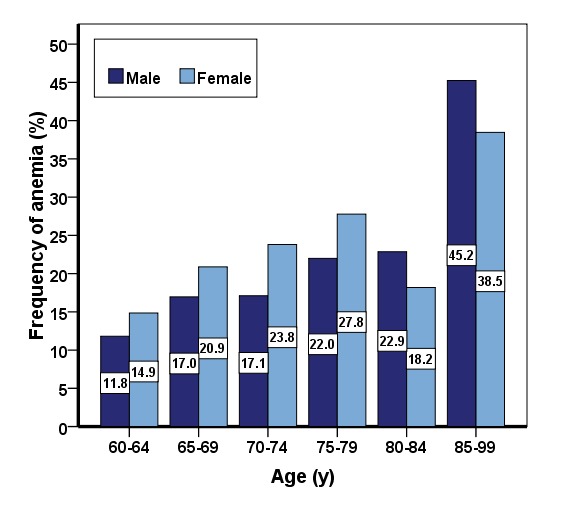
Frequency and percent of older people with anemia in Amirkola city according to their gender and age group

Overall, 1589 older adults including 864 men (54.4%) and 725 women (45.6%) were investigated. The mean age of the participants was 69.38 ±7.44 yr (range from 60 to 92 yr) and most of the people were in the 60 to 64 yr age group. The prevalence of anemia in older adults of Amirkola was 19.0% (302 participants). The overall prevalence of anemia was 20.3% among women and 17.9% among men. Moreover, the prevalence of anemia increased by age (*P*<0.001) (Figure1).

23.2% of participants with depressive symptoms had anemia, while this amount was 15.8% in participants without depressive symptoms (*P*<0.001).

Based on the GDS results, 895 participants were normal, 436 were mildly depressed, 176 were moderately depressed and 82 of them severely depressed. Overall, 694 participants (43.7%) had depressive symptoms.

The mean age of participants with and without depressive symptoms was 69.59 ±7.45 and 69.22 ±7.43, respectively. The highest rate of people with depressive symptoms was in 70-74 yr age groups (Table 1).

**Table 1:** Socio-demographic characteristics of the older people with and without depressive symptoms in the city of Amirkola

**Variables**	**Depressive symptoms**				***P*** **value**
	**Positive**		**Negative**		
	**Number**	**Percent**	**Number**	**Percent**	
Gender					0.001
Male	256	29.6	608	70.4	
Female	438	60.4	287	39.6	
Living alone					0.001
Yes	67	62.6	40	37.4	
No	627	42.3	855	57.7	
Smoking					0.068
Yes	114	38.9	179	61.1	
No	580	44.8	716	55.2	
Level of education					0.001
Illiterate	486	47.5	538	52.5	
Elementary and middle school	186	40.5	273	59.5	
High school and university	22	20.8	84	79.2	
Anemia					0.001
Positive	161	53.3	141	46.7	
Negative	533	41.4	754	58.6	
Age (yr)					0.100
60-64	242	42.9	322	57.1	
65-69	129	39.2	200	60.8	
70-74	139	50.0	139	50.0	
75-79	115	46.2	134	53.8	
80-84	44	38.6	70	61.4	
85-99	25	45.5	30	54.5	
Body mass index (Kg/m^2^)					0.011
<25.0	194	39.0	304	61.0	
25.0-29.9	277	42.7	371	57.3	
≤30.0	184	49.1	191	50.9	
Diabetes					0.001
Positive	246	51.9	228	48.1	
Negative	422	39.4	650	60.6	
Hypertension					0.026
Positive	441	45.4	531	54.6	
Negative	227	39.5	347	60.5	
Mental State Examination					0.001
Normal	396	36.4	691	63.6	
Abnormal	297	59.4	203	40.6	

In the group with depressive symptoms, the mean hemoglobin, hematocrit and serum iron level was lower and the mean TIBC was higher than the group without depressive symptoms but there was no significant difference in the ferritin level of the two groups. Moreover, the average number of chronic diseases in participants with depressive symptoms was significantly higher than participants without depressive symptoms (Table 2).

**Table 2:** The mean and standard deviation of effective variables on depressive symptoms among older adults of Amirkola

**Variables**	**Depression**	**Number**	**Mean**	**SD**	***P *** **value**
Age (yr)	Positive	694	69.59	7.45	0.320
	Negative	895	69.22	7.43	
Hemoglobin(gr/dl)	Positive	694	13.29	1.63	0.001
	Negative	895	13.96	1.50	
Hematocrit (%)	Positive	694	40.71	4.48	0.001
	Negative	895	42.38	4.03	
Serum Iron (μg/dL)	Positive	682	79.83	34.54	0.001
	Negative	888	86.56	36.34	
Total Iron Binding Capacity (μg/dL)	Positive	682	285.95	40.69	0.001
	Negative	889	279.23	38.38	
Ferritin (ng/ml)	Positive	686	168.78	125.81	0.260
	Negative	888	161.64	122.84	
Chronic Diseases	Positive	694	3.47	2.04	0.001
	Negative	894	2.15	1.66	
Mini Mental State Examination	Positive	693	24.13	4.47	0.001
	Negative	894	26.04	3.22	
Body mass index (Kg/m^2^)	Positive	655	27.53	4.75	0.020
	Negative	866	26.97	4.44	
Social support	Positive	694	26.41	3.60	0.001
	Negative	895	28.30	2.80	

**Figure 2:**
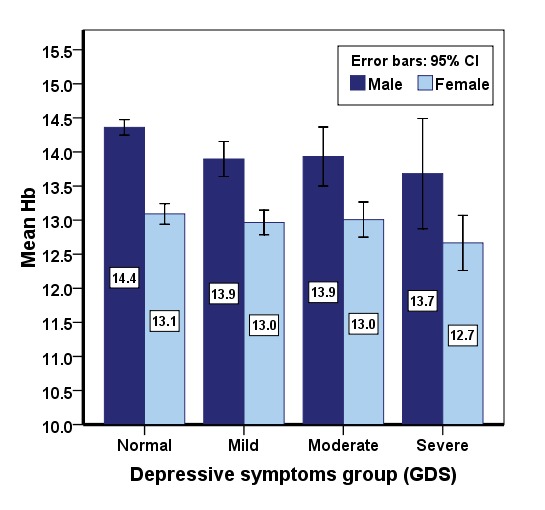
Mean hemoglobin level by depressive symptoms group and gender among older adults of Amirkola

According to logistic regression model, depressive symptoms were most highly related to gender, smoking, Mini-Mental State Examination (MMSE), diabetes and anemia (Table 3). In Figure 2, the mean hemoglobin level decreases with the increase in severity of depressive symptoms.

**Table 3:** Odds ratios and confidence interval of variables affecting on depression symptoms in raw and adapted conditions based on the logistic regression model in the elderly of Amirkola.

**Variables**	**Crude OR (CI 95%)**	***P*** **value**	**Adjusted OR** ^*^ **(CI 95%)**	***P*** **value**
Anemia (yes/no)	1.62 (1.26, 2.08)	0.001	1.46 (1.09, 1.95)	0.010
Diabetes (yes/no)	1.66 (1.34, 2.07)	0.001	1.51 (1.18, 1.92)	0.001
Hypertension (yes/no)	1.27 (1.03, 1.57)	0.026	1.07 (0.85, 1.36)	0.554
Mini mental state examination (abnormal/normal)	2.55 (2.06, 3.17)	0.001	1.82 (1.39, 2.38)	0.001
Gender/Female	1.61 (1.27, 2.08)	0.001	3.67 (2.80, 4.81)	0.001
Smoking (yes/no)	0.79 (0.61, 1.02)	0.069	1.92 (1.40, 2.64)	0.001
Living alone (yes/no)	2.28 (1.52, 3.42)	0.001	1.53 (0.97, 2.42)	0.067
Level of Education				
Illiterate	1.00		1.00	
Elementary and middle school	0.75 (0.60, 0.94)	0.013	0.94 (0.72, 1.22)	0.640
High School and University	0.29 (0.18, 0.47)	0.001	0.60 (0.35, 1.02)	0.060
Age (yr)				
60-64	1.00		1.00	
65-69	0.86 (0.65, 1.13)	0.280	0.77 (0.56, 1.05)	0.097
70-74	1.33 (0.99, 1.77)	0.052	1.36 (0.97, 1.91)	0.071
75-79	1.14 (0.85, 1.54)	0.390	0.94 (0.65, 1.35)	0.720
80-84	0.84 (0.55, 1.26)	0.390	0.64 (0.38, 1.07)	0.088
85-99	1.11 (0.64, 1.94)	0.720	0.67 (0.33, 1.35)	0.260
Body mass index (Kg/m^2^)				
<25.0	1.00		1.00	
25.0-29.9	1.17 (0.92, 1.48)	1.960	1.06 (0.81, 1.39)	0.660
≤30.0	1.51 (1.15, 1.98)	0.003	0.93 (0.68, 1.29)	0.680

*Adjusted for Gender, smoking, living alone, level of education, age, anemia,BMI, Diabetes, hypertension and MMSE.

## Discussion

The prevalence of Anemia in this study was 19.0% (302 participants), of which 51.3% (155) were male and 48.7% (147) were female. This amount is higher than other studies^17,18,19^ which is probably because of nutrient deficiencies, hemorrhage or chronic diseases. Moreover, the overall prevalence of anemia was 20.3% in women and 17.9% in men which is probably due to different demographic, social, cultural and nutritional conditions. Moreover, anemia was more prevalent among older adult women compared to men because of their several pregnancies and not receiving enough supplements. In Turkey, the prevalence of anemia in the elderly was 7.3%, with the prevalence of anemia in men (9.2%) more than in women (5.3%)^17^. In Pakistan, 31.0% of the elderly were anemic^20^. In the United States, 11.0% of men and 10.0% of women aged 65 yr and older suffer from anemia and this amount doubles among people over 85 yr old ^18^. In England, the prevalence of anemia was 5.2% ^19^ which was lower than the present study.

In a systematic review, the overall prevalence of anemia was 17.0%. Moreover, the prevalence of anemia was slightly higher in men (15.0%) than in women (14.0%) ^6^, which is contrary to the findings of the present study. In Korea, the prevalence of anemia in older adults was 13.3% associated with female gender, being a non-smoker, a low BMI and a low income and was more prevalent in people with chronic diseases such as depression, hypertension, diabetes, and dementia^21^. Anemia in older adults was associated with age, BMI, hypertension, renal failure, dementia, depression and disability ^22^. Undoubtedly, along with costliness and negative effects on health, chronic diseases have important roles in development and progression of disability in older adults. Therefore, interventions for management, prevention, and reduction of the probable constraints are essential.

The results of the present study showed that there was a direct relationship between anemia and depressive symptoms and after adjusting the variables this relationship still existed. Anemia can have unpleasant effects on older adults’ quality of life by causing symptoms including fatigue, irritability and poor concentration, which are symptoms of depression, and by decreasing the amount of oxygen reaching the brain which affects the brain’s performance^23^. Fatigue, which is a common symptom in chronic diseases such as anemia, can have destructive effects on people’s quality of life and lead to onset of depressive symptoms^24^. Moreover, a low hemoglobin level can be due to a series of inadequate dietary intake of iron which happens with depressed mood more often^23^. In a study on Italian older adults, the older adults with mild anemia had significantly worse results in cognitive, functional and mood assessments^25^ which were in line with the findings of the present study. In Seoul, South Korea, the relationship between anemia, subcortical ischemic changes and depressive symptoms was investigated and showed that anemia with severe subcortical ischemic changes was associated with depressive symptoms in patients with mild cognitive impairment^26^. In China, there was a direct relationship between low hemoglobin concentration and depression^23^. The findings of our study indicated the same relationship.

In the present study, depression among older adults was significantly associated with female gender, life alone, low levels of education, diabetes, hypertension and the MMSE score. There was a significant relationship between marital status and depressive symptoms among older adults in such a way that widowed older adults had more depressive symptoms than married older adults^27^. As a result, friends and families support older adults. Cognitive impairment, functional disability and chronic diseases were significantly related to increased GDS score and depressive symptoms ^28,29^. The overall prevalence of depression among older adults was 10.5%, not significantly related to gender but it was significantly associated with age groups and the highest risk factors were lack of social engagement, low family support, chronic disease and disturbed sleep^30^. Therefore, the healthcare personnel and community health nurses have to provide appropriate interventions during their home visits, especially for families of older adults and to educate in creating a safe and secure environment.

In Japan, people with higher GDS scores had considerably lower levels of hemoglobin compared to people with lower GDS scores and low hemoglobin level was significantly associated with depressed mode in women^31^. In a study on older adults with nutrient deficiency, depressive symptoms were only significantly related to low hemoglobin count and vitamin B6 deficiency. Moreover, older adults with coexisting deficiencies of nutrients contributing to production of hemoglobin were more likely to undergo depressed mood and emotions and their effects on daily activities^32^.

Iron deficiency and low level of serum ferritin have been associated with depression symptoms^33^. In another study, older people with depression had a lower serum folate level (21.5 nmol/L) compared to those without depression (24.0 nmol/L) and there was a linear relationship between decreased folate concentration and increased risk of developing depressive symptoms, independent of other risk factors including demographic, chronic diseases, nutritional risk, albumin, anemia and vitamin supplements such as vitamin B12, homocysteine etc. In addition, vitamin B12 deficiency was significantly related to depressive symptoms^34^. In order to prevent anemia and depression in older adults of the society, it is a necessity for healthcare experts to give them the required training to adhere to a healthy and nutritious diet.

The present study was a cross-sectional research, while investigation of the causes and effects of the association between anemia and depressive symptoms or vice versa requires a cohort study. Moreover, many factors can affect anemia including gastrointestinal, pulmonary, inflammatory and chronic diseases, type of diet, micronutrient deficiency and drugs such as aspirin and NSAIDs, not considered in our study. In some studies^32,34^ certain types of anemia were found to be effective on depression but we did not investigate different types of anemia.

Our study was population-based and it was conducted with a high participation rate among older adults of the city of Amirkola. It provided an accurate estimate of prevalence of anemia among this population regarded as strength of this study. In order to find the causal relationship between anemia and depressive symptoms, we suggest cohort studies to be conducted to investigate other effective factors on these illnesses.

## Conclusion

The present study showed the high prevalence of anemia and its direct relationship with depressive symptoms in older adults, which reflects the need for an accurate and sensitive screening among this vulnerable population. Since various environmental factors and the lifestyle had a crucial role in occurrence of these diseases, and regarding the growing population of older adults and the considerable rates of illiteracy among them, preventive interventions for these diseases have to become a priority and planning to reach the goal of having healthy older adults in the society as well as making decisions to adopt a healthy lifestyle, especially in the final years of life, should be taken seriously. Moreover, devising plans to improve the older adults’ awareness of prevention, timely diagnosis, and early interventions can limit the abovementioned illnesses.

## Conflict of interest statement

The authors declare that they have no conflict of interest.

## Funding

This work was funded by deputy of research and technology at Babol University of Medical Sciences.

## Acknowledgements

We thank the staff at number one and number two healthcare centers in the city of Amirkola for their cooperation in this plan and the honorable older adults of Amirkola for their participation.

## Highlights

- The prevalence of anemia and depression in the elderly was 19% and 43.67%, respectively.
- The overall prevalence of anemia was 20.3% among women and 17.9% among men.
- 23.2% of the elderly with symptoms of depression were anemic, while in participants without symptoms of depression it was 15.8%.
- Mean serum hemoglobin, hematocrit and iron in the group with depression symptoms was less than the group without symptoms of depression
